# CT screened arterial calcification as a risk factor for mortality after trauma

**DOI:** 10.1186/s13049-016-0317-1

**Published:** 2016-10-10

**Authors:** Henry D. De’Ath, Kathryn Oakland, Karim Brohi

**Affiliations:** Centre for Trauma Sciences, Blizard Institute, Barts and the London School of Medicine, Queen Mary University of London, Newark Street, London, E1 2AT UK

**Keywords:** Coronary artery diseases, Cardiac imaging and diagnostics, Aortic and arterial disease, Trauma, Outcomes

## Abstract

**Background:**

Arterial calcification on Computerised Tomography (CT) is a marker of cardiovascular disease. It is predictive of future adverse cardiac events and mortality in many disease states. The incidence of arterial disease and its impact on outcomes of the injured is not known. The objectives of this study were to describe the incidence of arterial calcification in trauma patients, and establish its impact on mortality.

**Methods:**

A retrospective cohort study of all injured patients aged over 45 years presenting to a major trauma centre over a 34-month period. The presence and quantity of coronary, aortic and abdominal arterial calcification on admission CT scans of the chest, abdomen and pelvis was established, and the association between cardiovascular disease and in-hospital mortality following trauma was determined.

**Results:**

Five hundred ninety-one patients were included in the study. Cardiac calcium was visible on 432 (73 %) scans, and abdominal arterial calcification on 472 (79.9 %). Fifty (8.5 %) patients died. Patients with Superior Mesenteric (SMA) and Common Iliac Artery calcification had a significantly higher mortality than those without (*p* < 0.01).

In multivariarate analysis, only SMA calcification was independently associated with mortality (OR 2.462, 95 % CI 1.08–5.60, *p* = 0.032). Coronary calcium demonstrated no independent statistical relationship with death (Left Anterior Descending Artery OR 1.189, 95 % CI 0.51–2.78, Circumflex OR 1.290, 95 % CI 0.56–2.98, Right Coronary Artery OR 0.483, 95 % CI 0.21–1.10).

**Discussion:**

This study has demonstrated that the identification of arterial calcification on admission CT scans of trauma patients is possible. Calcification was common, and present in around three-quarters of injured individuals over the age of 45 years. SMA calcium was an independent predictor of mortality. However, whilst the presence of arterial calcium demonstrated a tendency towards lower survival, this association was not significant in other territories, including the coronary arteries. Future studies should investigate further the association and pathophysiology linking SMA disease and mortality in trauma, in addition to the relationship between longer tem survival, adverse cardiac events and arterial calcification in injured patients.

**Conclusions:**

Arterial calcification can be reliably identified on trauma CT scans, and is common in injured patients. Abdominal vascular calcification appears to be a better predictor of mortality than coronary artery disease.

**Electronic supplementary material:**

The online version of this article (doi:10.1186/s13049-016-0317-1) contains supplementary material, which is available to authorized users.

## Background

In injured patients, both the presence and number of co-morbidities are associated with higher mortality [1]. The effect of pre-existing cardiovascular disease on survival following trauma has been the subject of few studies however, and its impact remains uncertain.

The limited research that has been undertaken has shown an association between pre-morbid heart disease and poorer survival [2], but there is also conflicting evidence suggesting that the presence of cardiac disease pre-injury has no influence on mortality [3, 4], or only has a limited effect in selective patient cohorts [5, 6].

The impact of cardiovascular disease on death in trauma has only been measured in those with a documented pre-injury diagnosis, and therefore symptomatic patients. A substantial number of injured individuals are likely to present with asymptomatic, subclinical disease, although studies have yet to investigate this group of patients. The true incidence of cardiovascular disease in the older trauma population is therefore unknown.

On computerised tomography (CT) scans of the chest, the presence of calcium in the coronary arteries is an indicator of subclinical coronary heart disease (CHD) [7], and directly correlates with atherosclerotic plaques on histological examination [8]. Furthermore, an absence of coronary artery calcium (CAC) tends to rule out luminal obstructive disease [9].

The objectives of this study, therefore, were to establish if arterial calcification could be reliably determined on emergent CT scans performed during the initial assessment of trauma patients, and if so, to measure the incidence of arterial calcification. Finally, to determine the association of underlying atherosclerosis with mortality after trauma. We hypothesised that the presence of arterial calcification would be associated with an increased mortality in trauma patients after injury.

## Methods

### Study aims, design and setting

The aims of this study were to establish if arterial calcification could be determined on emergency trauma CT scans, and if so, used to identify the incidence of calcification and its association with mortality after injury.

A single centre retrospective cohort study performed at a major trauma centre in the United Kingdom.

### Study population

All trauma patients aged 45 years or over who presented to the hospital over a 34-month period were eligible for enrolment. Individuals were identified retrospectively from the trauma registry, a prospectively collected database on every injured patient presenting to the hospital since 2003.

The age selection of 45 was based on established cardiovascular risk prediction algorithms and evidence on the point at which age has a negative impact on outcome in trauma [10]. Patients without a CT scan of the chest, abdomen or pelvis or incomplete records were excluded.

### Indication for CT in study patients

Scans were performed as part of the initial assessment of patients following trauma. CT was indicated in patients who presented with severe injuries, abnormal physiology or with a significant traumatic mechanism.

The trauma team who were independent to this study requested all scans. All CTs were undertaken within 3 hours of arrival to the Emergency Department.

### CT scan protocol in trauma

All CT scans were performed on a Siemens SOMATOM Sensation 64 slice scanner (Siemens Medical Solutions, Forchheim, Germany).

Patients were placed supine and entered the scanner head first with arms above their heads whenever possible. The scans covered the lung apices to the inferior aspect of the pelvis.

The delay between the start of contrast medium administration and the start of scanning was obtained using an automated bolus triggering technique (CARE bolus, Siemens medical solutions). In all patients, 80 mls of Visipaque contrast was used (Visipaque 270, GE Healthcare, Milwaukee, WI, USA) and injected at a rate of 2–3 mls/s with a scan delay of 50 s.

Data were acquired in a cranio-caudal direction with the following scanning parameters: 120 kVP tube voltage, 260 mAs, collimation 24 × 1.2 mm, 0.5 s rotation time and 3 mm increment and 3 mm reconstructed section thickness. A smooth reconstruction thickness (B31f) was used for initial reconstruction, followed by reconstructions using the settings of soft tissue fine, lung, bone fine, coronal lung, coronal thorax and abdomen, coronal spine/pelvis and sagittal spine.

### CT scan interpretation

Images were retrospectively analyzed for the presence of calcium, defined as 3 contiguous pixels of ≥130 Hounsfield Units [11]. The first authors reviewed each CT scan independently and determined either the presence or absence of calcification in the coronary arteries (left anterior descending [LAD], circumflex [Cx] and right coronary artery [RCA]), the thoracic and abdominal aorta, the coeliac axis, the superior mesenteric (SMA), inferior mesenteric (IMA) and common iliac arteries (CIA).

We further attempted to quantify the amount of coronary artery calcium, and thereby qualify the extent of cardiac calcification on survival in trauma. Following a validation exercise (Additional file 1: Supplement 1), each investigator examined every CT of the chest and ascribed both a numeric score (estimated coronary artery calcium score [CACS]) based on the quantity of calcium in the coronary arteries and a grade. The calcium score dictated the grade awarded (0 = none, 1–100 = mild, 101–400 = moderate, 401–1000 = severe and 1001 ≤ extensive). This was based on the American College of Cardiology Foundation/American Heart Association guidelines [12]. The number (0–3) and identity of vessels containing calcium were also documented.

Interpretation of scans was performed with observers blind to all patient characteristics, outcomes and each other’s measurements until assessment of the CT scans was complete in its entirety.

### Data collection

Data on patient demographics and baseline physiology, injury characteristics, injury severity score (ISS) and mortality were collected from the hospital trauma registry and from individual patient clinical records.

### Study outcome

The study endpoint was death, defined as death of any cause occurring in-hospital. For the purposes of analysis, patients surviving to hospital discharge were assumed to have survived.

### Statistical analysis

All data analyses were performed using SPSS version 20 (SPSS Inc., Chicago IL). Normal-quartile plots were used to test for normality. Non-parametric continuous data are reported as median with interquartile range and categorical data as absolute number and percentage. Mann-Whitney U or Kruskal-Wallis tests were used to compare numerical data and Fisher’s exact test or chi-square was used to compare categorical data.

Kaplan-Meier survival curves were created for each vascular bed and differences were analyzed using log rank (Mantel-Cox) analysis.

A binary logistic regression model was created to identify the independent effect of individual arterial beds on death in this patient cohort. The model contained both categorical and continuous predictor variables, and variables were added in a stepwise regression analysis. Significance levels were set at *p* < 0.05 to enter and *p* > 0.1 for removal.

Kappa coefficient (κ) was used to test inter-observer reliability for nominal data and Bland-Altman plots to interpret inter-observer agreement for continuous data (Additional file 1: Supplements 1 and 2).

## Results

Over the study period, 1385 injured patients aged 45 years or older were identified. Of these, 734 (53 %) did not undergo CT and were excluded. A further 16 (1.2 %) scans of the chest, abdomen and pelvis were incomplete and therefore omitted.

### Feasibility of trauma CT in determining calcified atherosclerosis

Of the 635 scans available, the interpretation of 44 (7.4 %) was not possible due to artefact from chest drains, other external devices or movement, and these patients were excluded from subsequent analyses. In just over 90 % of trauma CT scans, therefore, an assessment of the presence of arterial calcium was feasible.

The investigators were unanimous in their agreement (κ = 1) in identifying those images that could and could not be further assessed for the purposes of this study. Both investigators noted it was not possible to reliably identify calcification in the IMA, and no further analyses of this artery were undertaken.

The results of the inter-observer agreement analyses revealed that the estimation of the calcium load on CT scans of the study patients was both reliable and reproducible (Additional file 1: Supplement 2).

### Study population

The study population comprised of some 591 patients. The majority were male and moderately injured. Characteristics are shown in Table 1.

**Table 1:** Study population characteristics

	All	No calcium	Calcium	*p* value
Number	591	85 (14)	506 (86)	
Male	474	65 (76)	409 (81)	0.377
Age, years, Median (IQR)	56 (49–66)	48 (46–51)	63 (54–74)	<0.001
Co-morbidities	329	52 (61)	277 (55)	0.289
Injury characteristics				
Blunt mechanism	539	76 (89)	463 (91)	0.535
ISS, Median (IQR)	13 (6–25)	9 (2–24)	14 (8–27)	0.001
Outcomes				
Hospital length of stay, days, Median (IQR)	11 (2–25)	6 (2–25)	13 (3–25)	0.106
Mortality	50	2 (2)	48 (9)	0.033

Data presented as number (percentage) unless otherwise stated

*ISS* injury severity score

Three hundred and twenty nine (55.7 %) patients presented with one or more documented medical co-morbidities. Of these, cardiovascular diseases were most common (Table 2).

**Table 2:** Incidence of documented patient co-morbidities

Diagnosis	Incidence
Cardiovascular	200 (33.8)
Hypertension	74 (12.5)
Diabetes	40 (6.8)
Non-insulin dependent diabetes	33 (5.6)
Insulin dependent diabetes	7 (1.2)
Hypercholesterolemia	21 (3.6)
Arrythmias	18 (3.1)
Permanent Pacemaker	6 (1.0)
Ischaemic Heart Disease	24 (4.1)
Myocardial Infarction	8 (1.4)
Angina	6 (1.0)
CABG	7 (1.2)
Stenting	2 (0.4)
Angioplasty	1 (0.2)
Valvular Heart Disease	5 (0.9)
Aortic Valve	2 (0.4)
Mitral Valve	3 (0.5)
Rheumatic Heart Disease	1 (0.2)
Heart Failure	1 (0.2)
Abdominal Aortic Aneurysm	7 (1.2)
Peripheral Vascular Disease	3 (0.5)
Neurological	36 (6.1)
Cerebrovascular accident	14 (2.4)
Epilepsy	15 (2.5)
Dementia	6 (1.0)
Parkinson’s Disease	1 (0.2)
Psychiatric	24 (4.1)
Respiratory	25 (4.2)
Chronic Obstructive Pulmonary Disease	10 (1.7)
Asthma	15 (2.5)
Malignancy	11 (1.7)
Prostate	5 (0.9)
Lung	2 (0.4)
Bowel	1 (0.2)
Breast	1 (0.2)
Ovary	1 (0.2)
Other	19 (3.2)
Chronic Kidney Disease	6 (1.02)
Anaemia	2 (0.34)
Diverticular Disease	6 (1.02)
Sickle Cell	1 (0.17)

Data presented as absolute number (%)

### Incidence of calcified atherosclerosis on trauma CT Scans

**Fig. 1:**
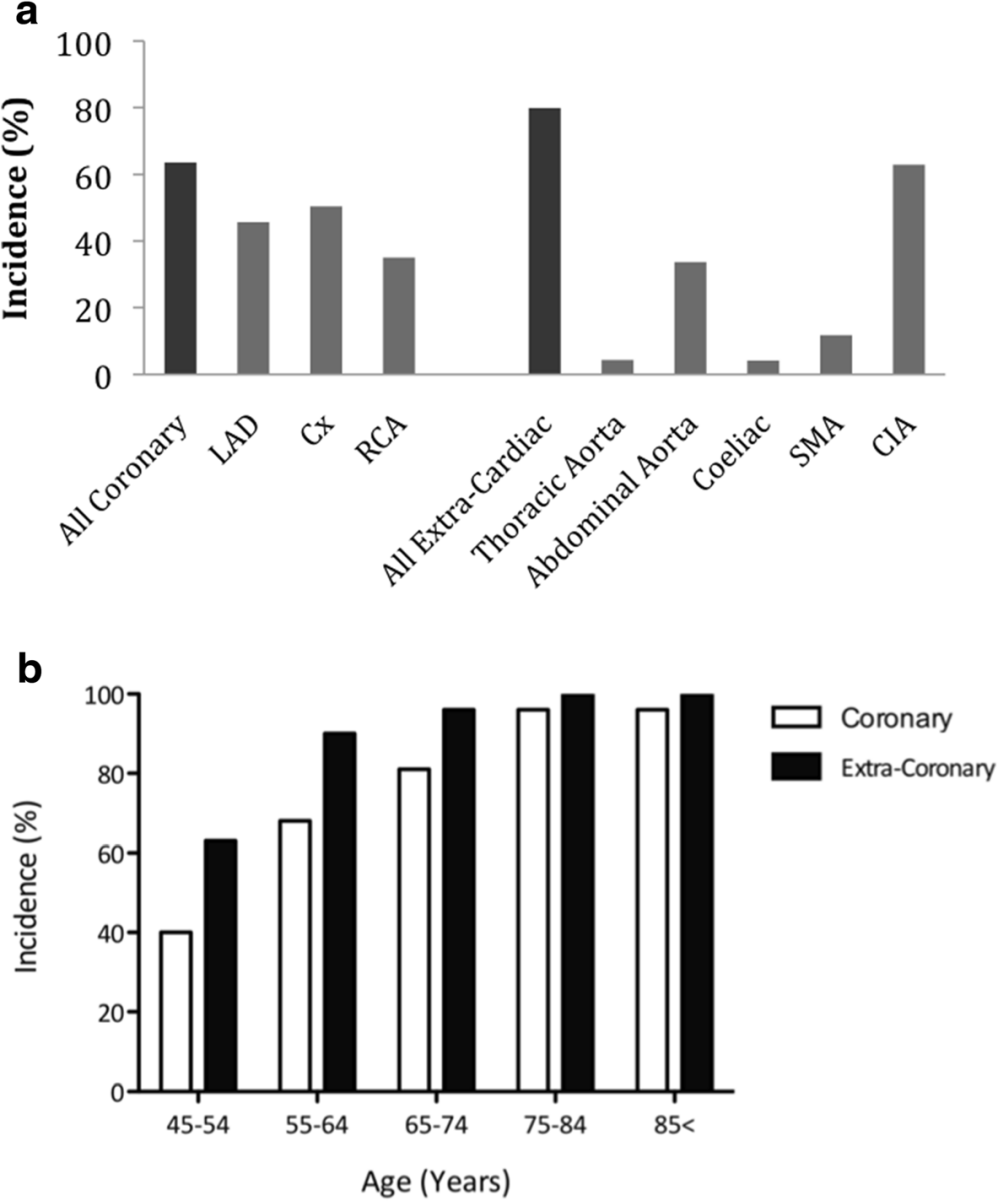
**a** Incidence Per Vessel of Calcified Atherosclerosis on Trauma CT Scans of Patients Aged ≥45 Years. **b** Incidence of Calcification by Age. The incidence of calcium increased significantly with age (coronary *p* < 0.001; extra-coronary *p* = 0.032)

Most patients had evidence of arterial calcification on admission trauma CT. Of the two broad territories, cardiac calcium was visible on 432 (73 %) scans, whilst extra-cardiac (abdominal) calcification was present on 472 (79.9 %). The most frequently calcified individual arterial bed was the CIA (Fig. 1a).

There was a significant trend towards an increasing incidence of calcium with advancing age, both in the cardiac (*p* < 0.001) and extra-cardiac (*p* = 0.032) regions (Fig. 1b).

### Calcification and mortality

**Fig. 2:**
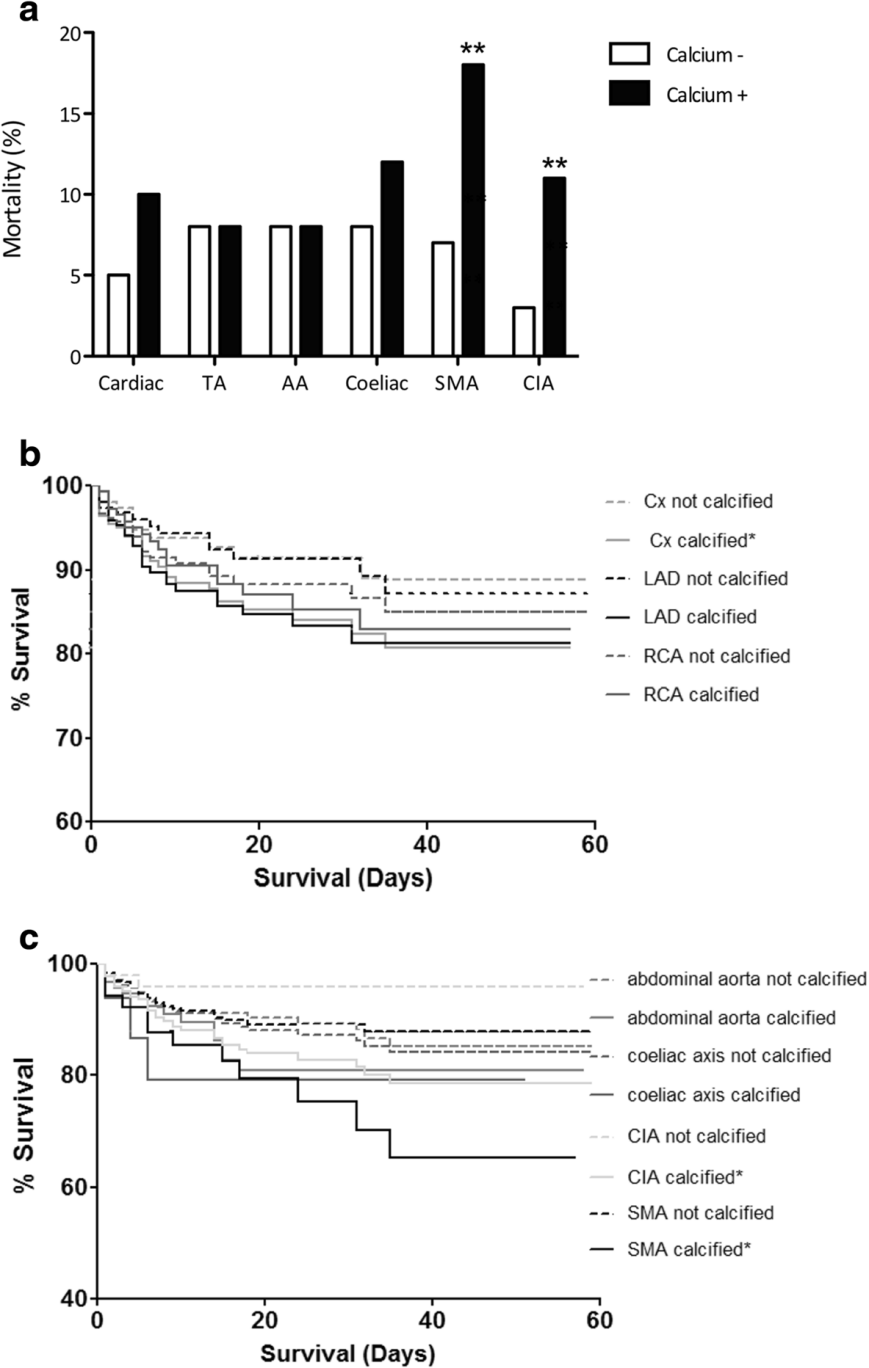
**a** Death Rate Based on the Absence or Presence of Coronary or Extra-Coronary Artery Calcium. TA = Thoracic Aorta, AA = Abdominal Aorta, SMA = Superior Mesenteric Artery, CIA = Common Iliac Arteries (**p < 0.01). **b** Kaplan-Meier Survival Curves for Coronary Vessels. LAD calcium *p* = 0.071, RCA calcium *p* = 0.764 and Cx calcium (* *p* < 0.05). **c** Kaplan-Meier Survival Curves for Extra-Coronary Vessels. AA calcium *p* = 0.345, Coeliac calcium *p* = 0.330, SMA calcium and CIA calcium (*p < 0.01)

Overall, 50 (8.5 %) patients died. Individuals with calcified atherosclerosis in the heart had a higher mortality rate compared to patients without, although differences were not significant (Fig. 2a). In the extra-cardiac territories, patients with calcification of the SMA (Odds Ratio 3.04, 95 % CI 1.53–6.07, *p* = 0.004) and CIA (Odds Ratio 3.36, 95 % CI 1.55–7.29, *p* = 0.001) had a significantly higher death rate than those without (Fig. 2a).

Although there was a trend towards poorer survival with calcification of individual coronary arteries, the curves for LAD and RCA calcium were not significantly different when compared to those without calcium in these vessels. However, calcification of the Circumflex artery was associated with lower survival (*p* < 0.05, Fig. 2b).

Survival curves for arteries in the abdominal territories also showed a trend towards reduced survival, but only SMA and CIA calcium were associated with significantly lower survival in comparison to those without calcification (*p* < 0.01). The survival curves for abdominal aorta and coeliac axis were not significantly different (Fig. 2c).

### Cardiac calcium severity and mortality

Characteristics of patients based on the CACS are shown in Table 3.

**Table 3:** Patient characteristics based on coronary artery calcium grade

	None	Mild	Moderate	Severe	Extensive
Number (%)	137 (31.2)	139 (32.2)	75 (17.4)	64 (14.8)	17 (3.9)
Age, years**	50 (47–56)	53 (45–89)	64 (45–94)	70 (46–91)	77 (58–91)
Gender, male *n* (%)	107 (78)	115 (83)	56 (75)	51 (80)	16 (94)
Co-morbidities, *n* (%)**	46 (33)	38 (27)	43 (57)	42 (66)	9 (53)
ISS	13 (2–25)	12 (5–28)	17 (9–29)	15 (9–27)	20 (3–32)
CACS**	0 (0–0)	45 (20–70)	190 (130–290)	617.5 (500–763)	1100 (1048–1150)
Vessels involved **	0 (0–0)	1 (1–2)	2 (2–3)	3 (3–3)	3 (3–3)
Hospital stay, days*	6 (2–25)	8 (2–20)	17 (5–30)	16 (4.5–25)	14 (1.5–48.5)

Data presented as median (interquartile range) unless otherwise stated. Comparisons are made across all groups. *p < 0.01, **p < 0.001

**Fig. 3:**
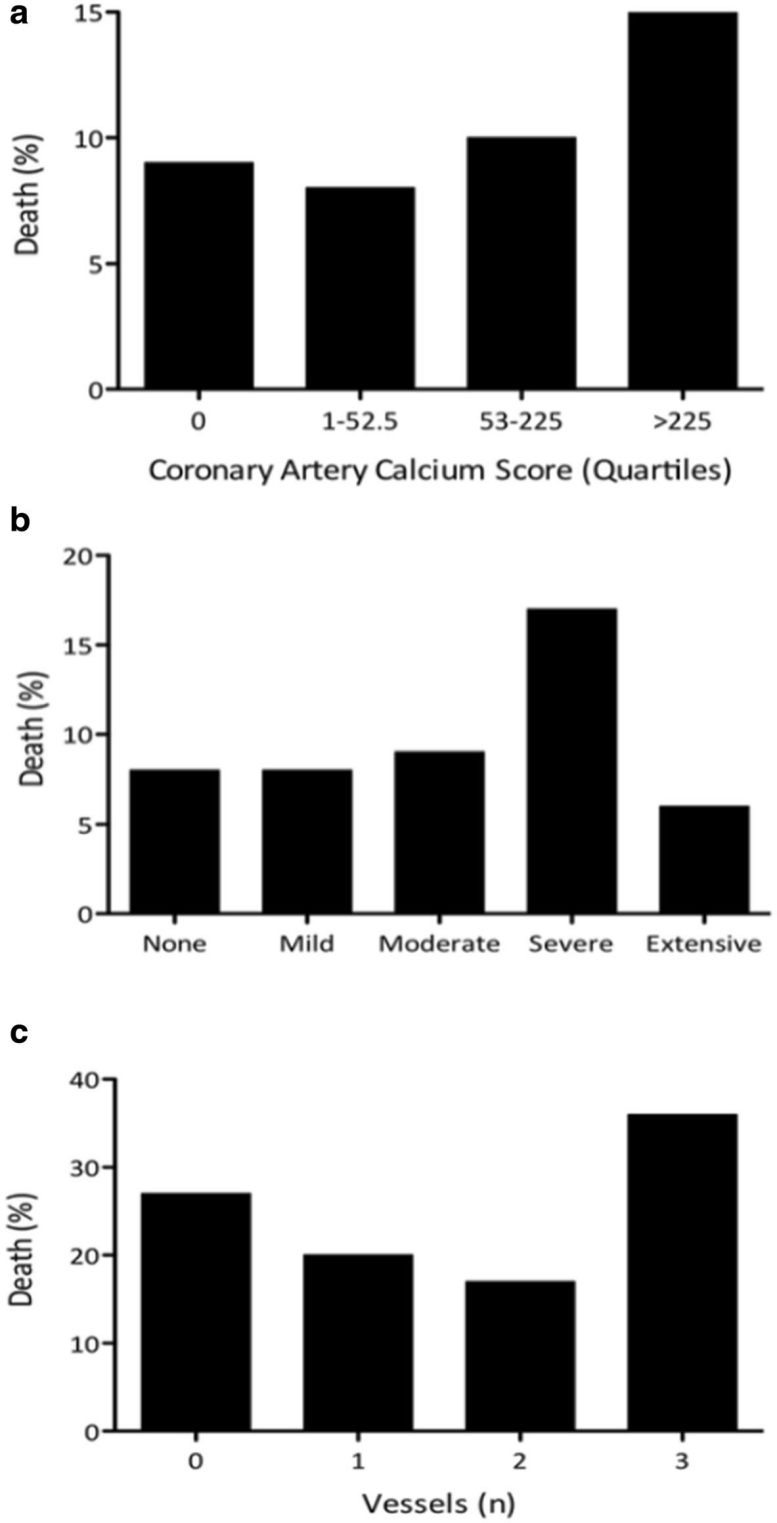
**a** Death Rate per Coronary Artery Calcium Score Quartile. There was a non-significant towards increasing death rates with higher CACS (*p* = 0.181). **b** Death Rate According to Grade of Coronary Artery Calcium. A grade of severe CAC had a higher death rate, though differences were not statistically significant (*p* = 0.157). **c** Mortality According to Number of Calcified Vessels Observed. Patients with three vessel disease had the highest incidence of death, although this was not significant (*p* = 0.173)

The death rate was highest in individuals in the highest CACS quartile, and there was a non-significant trend towards poorer survival with increasing calcium score (Fig. 3a).

Only a grade of severe CAC on admission trauma CT was associated with a higher death rate. Those with otherwise mild, moderate or extensive CAC were found to have no significant differences compared to patients without calcification (Fig. 3b).

Patients with calcification in all three coronary arteries had the highest incidence of death, but this was not statistically significant (Fig. 3c).

### Predictors of mortality in an older trauma cohort

In order to identify the clinical variables independently associated with death in this study population, a binary logistic regression model was created (Table 4).

**Table 4:** Predictors of death in trauma patients over the age of 45

Variable	Odds ratio	95 % CI	*p* value
Age	1.035	1.00–1.07	0.050
Sex (male)	0.323	0.16–0.67	0.003
ISS	1.073	1.05–1.10	0.000
Mechanism (blunt)	0.786	0.10–6.44	0.822
SMA	2.462	1.08–5.60	0.032
Common iliac	2.447	0.92–6.52	0.073
Coeliac	1.815	0.47–7.07	0.391
Abdominal aorta	1.626	0.77–3.42	0.201
Cx calcium	1.290	0.56–2.98	0.552
LAD calcium	1.189	0.51–2.78	0.689
RCA calcium	0.483	0.21–1.10	0.083

The *R* ^2^ value of the model was 0.253 (Nagelkerke R Square). Thoracic aorta calcification was excluded from the analysis due to such small numbers

Four predictors of death were identified and included age, ISS, sex and SMA calcification. Based on this regression analysis, the SMA territory was the only arterial bed independently associated with mortality (Table 4). The presence of SMA calcification was associated with a nearly 2.5 times increased odds ratio of in-hospital death (OR 2.462, 95 % CI 1.08–5.60, *p* = 0.032).

## Discussion

This study has demonstrated that the identification of arterial calcification on admission CT scans of injured patients is possible. Calcification was common, and around three-quarters of trauma patients over the age of 45 years displayed evidence of atherosclerotic cardiovascular disease. The presence of arterial calcium was associated with a tendency towards lower survival, although an increase in the distribution and severity of arterial calcification was not generally associated with in-hospital mortality following injury.

Establishing the prevalence of cardiovascular disease in trauma was previously limited by difficulties in identifying asymptomatic individuals. In the general population, multiple studies have demonstrated an association between high coronary artery calcium scores (CACS) with an increased risk of adverse cardiac events, both in symptomatic and subclinical heart disease [13–17]. CACS is predictive of all-cause mortality in patients with heart disease [18, 19], and provide cardiac screening assessments in other conditions including HIV [20] and aortic aneurysms [21]. Calcification of the abdominal aorta may be an independent predictor of cardiovascular events [22], whilst calcium in the thoracic aorta, carotids and iliac arteries on CT has been associated with increased long term mortality in healthy subjects [11]. Arterial calcification on CT is therefore likely to represents a suitable marker of cardiovascular disease in trauma patients.

Isolated levels of CAC were not generally predictive of survival and only patients with severe CACS had higher death rates. Some previous studies have shown an association between cardiac co-morbidities with poor outcome following trauma [1–3]. Given the incidence of cardiovascular disease within the study cohort, the lack of significance of association of coronary calcification with mortality was unexpected. Previous studies, however, have examined injured patients with symptomatic heart disease whereas our study population also included asymptomatic subjects. This might suggest it is only symptomatic heart disease which leads to poorer outcome in trauma.

In contrast, an independent association of SMA calcium with mortality was demonstrated. CIA calcification also showed a tendency towards increased mortality. CIA was the most frequently calcified vessel, however, and may simply be a marker of advanced systemic calcification. This has been suggested in research in non-trauma patients and an association between CIA calcium and long-term mortality has been revealed in healthy subjects [11]. Conversely, SMA calcification was rare, perhaps suggesting SMA disease is more discriminatory than that of the CIA, and that the location of calcification is related to outcome.

The relationship between arterial calcification and all-cause mortality as opposed to cardiovascular mortality is not understood. An association with higher levels of inflammatory mediators leading to a chronic inflammatory state has been suggested [23] whilst atherosclerosis may be linked with reduced innate and acquired immune function [24]. Mesenteric ischaemic reperfusion injury is known to play a role in the development of the systemic inflammatory response syndrome (SIRS) [25] and the number of SIRS variables present on admission predicts mortality in trauma [26]. It is possible that SMA atherosclerosis increases the ischaemia-reperfusion injury from the GI tract after trauma leading to increased incidence or severity of organ dysfunction. To this end, arterial stiffness is known to lead to end organ damage and poorer outcomes [27]. Research has revealed markers indicative of arterial stiffness (including central pulse pressure and carotid-femoral pulse wave velocity) have a positive association with both thoracic and aortic calcification [28]. Consequently, this detrimental relationship between adverse arterial haemodynamics, atherosclerosis and end organ perfusion may be a factor in the underlying pathophysiology of our findings. Nevertheless, the role of mesenteric atherosclerosis and arterial stiffness in post-traumatic inflammation and organ dysfunction require further study, including the use of echocardiography to examine whether left ventricular changes resulting from adverse arterial haemodynamics are seen in patients with increased SMA calcification.

This study is subject to limitations. Firstly, its retrospective nature may have led to inaccurate documentation of patient characteristics and outcomes, and restricted the availability of wide datasets and clinical features. In particular, a smoking history was not possible to ascertain, in spite of the potential significance of this information. Conclusions were derived from the presence of absence of calcium, or score estimates, but not objectively calculated values. Although it was an aim of this study to determine whether calcium score assessments could be made and applied, it means that the findings can only be generalised. However, formal calcium scoring of non-cardiac CTs is not possible, and so it would have been unfeasible to objectively measure calcium scores in this study. Our analyses were also limited to a single centre and may have been underpowered given few numbers in certain sub-groups. Finally, evidence on the prognostic value of arterial calcification on mortality has often followed up patients over many years. It would be valuable in future studies, therefore, to analyse whether arterial calcification is more predictive of the higher longer-term death rates seen in trauma patients.

## Conclusions

Trauma CT scans performed on admission in older injured patients may be used to estimate the burden of arterial calcification. These estimates suggest cardiovascular disease in this population is common. Calcification in the SMA was independently associated with an increased risk of in-hospital mortality. The presence and extent of coronary artery calcium is not, however, generally associated with in-hospital mortality after injury.

## Additional file

Additional file 1: Supplement 1. Training and Validation Exercise. Supplement 2. Study Inter-observer Agreement. (DOCX 169 kb)

## Abbreviations

CAC: Coronary artery calcium
CACS: Coronary artery calcium score
CHD: Coronary heart disease
CI: Confidence interval
CIA: Common iliac arteries
CT: Computerised tomography
Cx: Circumflex
IMA: Inferior mesenteric
ISS: Injury severity score
kVp: Peak kilovoltage
LAD: Left anterior descending
mA: Milliamps
OR: Odds ratio
RCA: Right coronary artery
SIRS: Systemic inflammatory response syndrome
SMA: Superior mesenteric

## References

[1] Bamvita J-M, Bergeron E, Lavoie A, Ratte S, Clas D (2007). The impact of premorbid conditions on temporal pattern and location of adult blunt trauma hospital deaths. J Trauma.

[2] Ferraris VA, Ferraris SP, Saha SP (2010). The relationship between mortality and preexisting cardiac disease in 5,971 trauma patients. J Trauma.

[3] Grossman MD, Miller D, Scaff DW, Arcona S (2002). When is an elder old? Effect of preexisting conditions on mortality in geriatric trauma. J Trauma.

[4] Smith DP, Enderson BL, Maull KI (1990). Trauma in the elderly: determinants of outcome. South Med J.

[5] Hollis S, Lecky F, Yates DW, Woodford M (2006). The effect of pre-existing medical conditions and age on mortality after injury. J Trauma.

[6] Shoko T, Shiraishi A, Kaji M, Otomo Y (2010). Effect of pre-existing medical conditions on in-hospital mortality: analysis of 20,257 trauma patients in Japan. J Am Coll Surg.

[7] Wexler LBB, Crouse J, Detrano R, Fuster V, Maddahi J, Rumberger J, Stanford W, White R, Taubert K (1996). Coronary artery calcification: pathophysiology, epidemiology, imaging methods, and clinical implications a statement for health professionals from the American Heart Association. Circulation.

[8] Rumberger JASD, Fitzpatrick LA, Sheedy PF, Schwartz RS (1995). Coronary artery calcium area by electron-beam computed tomography and coronary atherosclerotic plaque area. A histopathologic correlative study. Circulation.

[9] Simons DBSR, Edwards WD, Sheedy PF, Breen JF, Rumberger JA (1992). Noninvasive definition of anatomic coronary artery disease by ultrafast computed tomographic scanning: a quantitative pathologic comparison study. J Am Coll Cardiol.

[10] Grundy SM, Pasternak R, Greenland P, Smith S, Fuster V (1999). Assessment of cardiovascular risk by use of multiple-risk-factor assessment equations: a statement for healthcare professionals from the American Heart Association and the American College of Cardiology. Circulation.

[11] Allison MAHS, Wassel CL, Morgan C, Ix JH, Wright CM, Criqui MH (2012). Calcified atherosclerosis in different vascular beds and the risk of mortality. Arterioscler Thromb Vasc Biol.

[12] Greenland PBR, Brundage BH, Budoff MJ, Eisenberg MJ, Grundy SM, Lauer MS, Post WS, Raggi P, Redberg RF, Rodgers GP, Shaw LJ, Taylor AJ, Weintraub WS, American College of Cardiology Foundation Clinical Expert Consensus Task Force (ACCF/AHA Writing Committee to Update the 2000 Expert Consensus Document on Electron Beam Computed Tomography); Society of Atherosclerosis Imaging and Prevention; Society of Cardiovascular Computed Tomography (2007). ACCF/AHA 2007 clinical expert consensus document on coronary artery calcium scoring by computed tomography in global cardiovascular risk assessment and in evaluation of patients with chest pain. J Am Coll Cardiol.

[13] Agatston AS, Janowitz WR, Hildner FJ, Zusmer NR, Viamonte M, Detrano R (1990). Quantification of coronary artery calcium using ultrafast computed tomography. J Am Coll Cardiol.

[14] Arad YSL, Goodman K, Lledo-Perez A, Sherman S, Lerner G, Guerci AD (1996). Predictive value of electron beam computed tomography of the coronary arteries. 19-month follow-up of 1173 asymptomatic subjects. Circulation.

[15] Arad YSL, Goodman K, Newstein D, Guerci AD (2000). Prediction of coronary events with electron beam computed tomography. J Am Coll Cardiol.

[16] Detrano RGA, Carr JJ, Bild DE, Burke G, Folsom AR, Liu K, Shea S, Szklo M, Bluemke DA, O’Leary DH, Tracy R, Watson K, Wong ND, Kronmal RA (2008). Coronary calcium as a predictor of coronary events in four racial or ethnic groups. N Engl J Med.

[17] Polonsky TS, McClelland RL, Jorgensen NW, Bild DE, Burke GL, Guerci AD, Greenland P (2010). Coronary artery calcium score and risk classification for coronary heart disease prediction. JAMA.

[18] Raggi P, Gongora MC, Gopal A, Callister TQ, Budoff M, Shaw LJ (2008). Coronary artery calcium to predict all-cause mortality in elderly men and women. J Am Coll Cardiol.

[19] Budoff MJ, Shaw LJ, Liu ST, Weinstein SR, Mosler TP, Tseng PH, Flores FR, Callister TQ, Raggi P, Berman DS (2007). Long-term prognosis associated with coronary calcification. J Am Coll Cardiol.

[20] d’Ettorre G, Francone M, Mancone M, Ceccarelli G, Ascarelli A, Vullo F, Baroncelli S, Galluzzo MC, Catalano C, Strano S, Fedele F, Mastroianni C, Palmisano L, Vullo V (2011). Significant coronary stenosis detected by coronary computed angiography in asymptomatic HIV infected subjects. J Infect.

[21] Stolzmann P, Phan C, Desbiolles L, Lachat M, Pfammatter T, Marincek B, Prokop M, Alkadhi H (2009). The heart of patients with aortic aneurysms: evidence from cardiac computed tomography. Interact Cardiovasc Thorac Surg.

[22] Criqui MHDJ, McClelland RL, Allison MA, Ix JH, Guerci A, Cohoon KP, Srikanthan P, Watson KE, Wong ND (2014). Abdominal aortic calcium, coronary artery calcium, and cardiovascular morbidity and mortality in the Multi-Ethnic Study of Atherosclerosis. Arterioscler Thromb Vasc Biol.

[23] Ross R (1999). Atherosclerosis--an inflammatory disease. N Engl J Med.

[24] Hansson GK, Libby P, Schonbeck U, Yan Z-Q (2002). Innate and adaptive immunity in the pathogenesis of atherosclerosis. Circ Res.

[25] Vollmar B, Menger MB (2011). Intestinal ischemia/reperfusion: microcirculatory pathology and functional consequences. Langenbeck’s Arch Surg.

[26] Malone DL, Kuhls D, Napolitano LM, McCarter R, Scalea T (2001). Back to basics: validation of the admission systemic inflammatory response syndrome score in predicting outcome in trauma. J Trauma.

[27] Safar ME, Nilsson PM, Blacher J, Mimran A (2012). Pulse pressure, arterial stiffness, and end-organ damage. Curr Hypertens Rep.

[28] Tsao CW, Pencina KM, Massaro JM, Benjamin EJ, Levy D, Vasan RS, Hoffmann U, O’Donnell CJ, Mitchell GF (2014). Cross-sectional relations of arterial stiffness, pressure pulsatility, wave reflection, and arterial calcification. Arterioscler Thromb Vasc Biol.

[29] Changes to the remit of Research Ethics Committees (2011). National Research Ethics Service.

