# Supplementary material for: CT screened arterial calcification as a risk factor for mortality after trauma
Source: Scand J Trauma Resusc Emerg Med. 2016 Oct 10;24:120. doi: 10.1186/s13049-016-0317-1 (PMC5057451; doi:10.1186/s13049-016-0317-1)
Supplement: Additional file 1: — Supplement 1. Training and Validation Exercise. Supplement 2. Study Inter-observer Agreement. (DOCX 169 kb) [file 13049_2016_317_MOESM1_ESM.docx]

**Additional file 1**

**Supplement 1**

**Training and Validation Exercise for Assessment of Coronary Artery Calcium Score and Grade**

This exercise was designed to train and validate the ability of the joint first authors to estimate the coronary artery calcium score (CACS) and grade.

In the first instance, the burden of calcium was examined on existing non-trauma CT scans with an established formal calcium score. This was followed by a validation assignment, whereby the investigators’ capacity to interpret and agree on the CACS was measured.

**Training**

Both investigators reviewed and familiarised themselves with the features of 200 CT scans of the heart with formal calcium scores (Agatston scores) performed in patients with known coronary atherosclerosis. These images were obtained by the hospital cardiology team and were undertaken independently for the purpose of assessment of the coronary arteries in patients with symptomatic heart disease. These 200 scans were selected at random from the list of patients found in the cardiology database.

The scans were undertaken using a second generation dual-source CT scanner (SOMATOM Definition Flash, Siemens, Forchheim, Germany). These heart scans were acquired with a prospectively ECG-triggered technique at 40% of the cardiac cycle when the patient's heart rate was higher than 75 beats per minute, otherwise with a high pitch spiral technique.

Scan parameters were as follows: collimation 0.6 mm, x-ray tube voltage 120 kilovolts (kV), x-ray tube current 250 milliamperes (mA). Three millimitre thick images were reconstructed at an increment of 1.5 mm.

MSCT datasets were analysed using a dedicated workstation (syngo MultiModality Workplace, Siemens, Erlangen, Germany) and specific software (syngo Calcium Scoring, Siemens, Germany) was used to measure calcium score on non-enhanced images.

**Validation**

Once familiar with the characteristics of the 200 formal CT calcium scores, a different set of 50 coronary CT scans were also selected at random from the database to validate the ability of each observer to accurately estimate a CACS.

These 50 scans had also been previously undertaken to evaluate non-trauma patients with symptomatic heart disease and each had already been ascribed a formal Agatston score. The two investigators assessed these scans independently, and whilst blinded to both one another’s estimates and the formal Agatston score.

Each investigator gave both a numeric score (estimated CACS) based on the quantity of calcium in the coronary arteries and a grade. Grades were awarded on the basis of the calcium score (Table 1), and were based on the 2010 American College of Cardiology Foundation/American Heart Association guidelines ([16](#_ENREF_16)).

**Table 1. Grades of Calcium Based on Corresponding Calcium Score.**

| **Calcium Grade** | **Calcium Score** |
| --- | --- |
| None | 0 |
| Mild | 1-100 |
| Moderate | 101-400 |
| Severe | 401-1000 |
| Extensive | ≥1001 |

**Inter-observer Agreement**

Agreement between the two observers was excellent for both grade (kappa coefficient κ=0.82) and score (Figure 1).


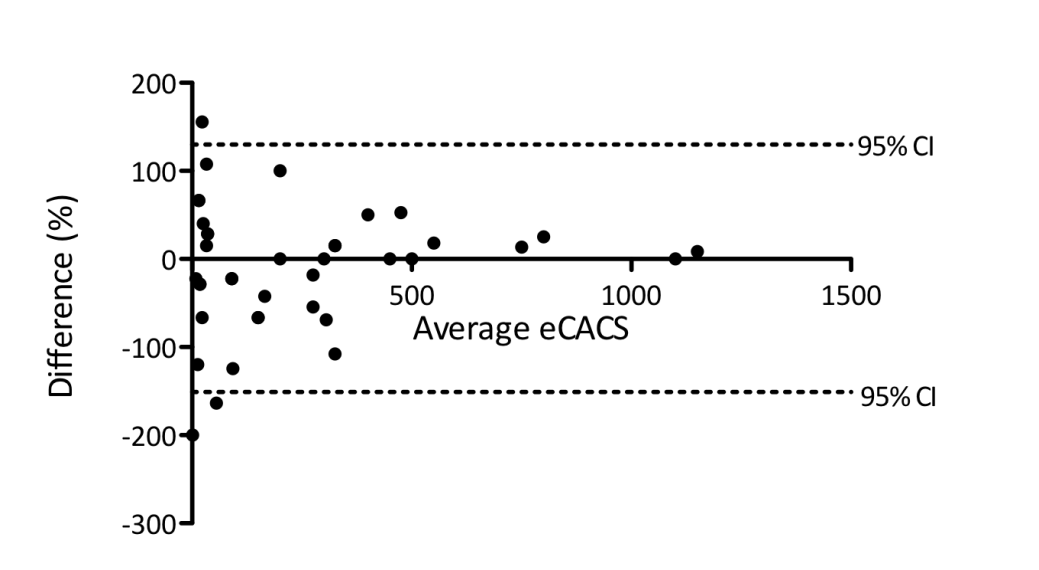


**Figure 1. Bland Altman plot of inter-observer agreement in validation exercise**. (Bias=-10.6, SD of bias 71.67).

Agreement between the first observer and grade based on the formal Agatston score was excellent (κ=0.80) and between the second observer and formal grade was good (κ=0.64).

**Supplement 2**

**Study Inter-observer Agreement**

There was substantial agreement between observers when analysing the presence or absence of calcium (κ=0.74).

When considering coronary artery calcium scores, there was good agreement between the CACS of the two observers with 92% of estimates falling within the 95% Confidence Intervals (Figure 1). Where there was disagreement, this was predominantly in patients with a lower CACS and as the score increased, so did the inter-observer concordance (Figure 1).


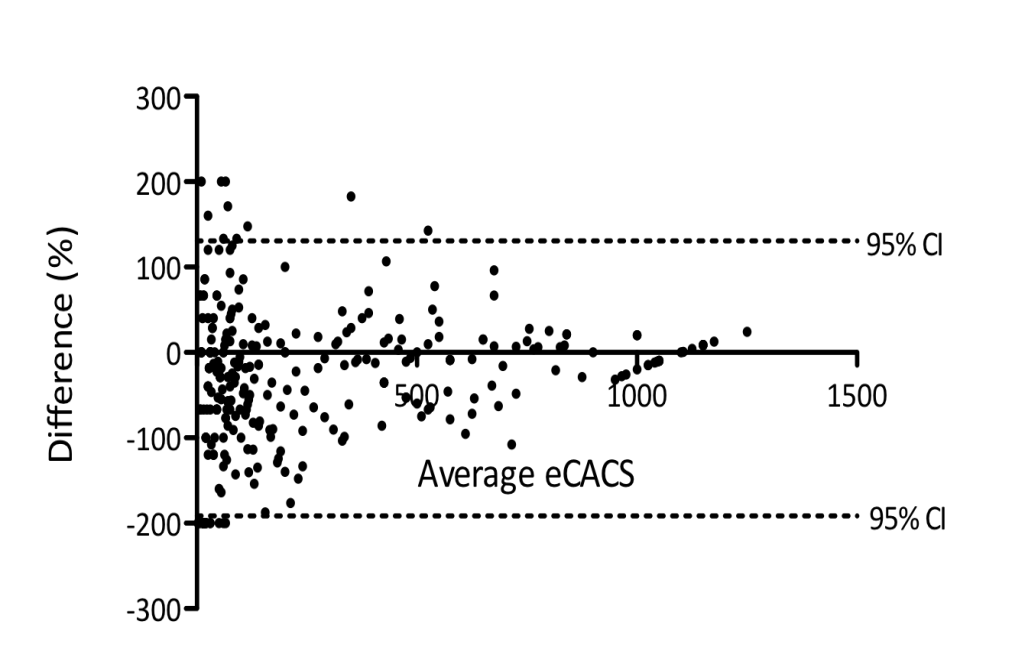


**Figure 1**. **Bland Altman Plot of Inter-Observer Agreement of Estimated Coronary Artery Calcium Scores**. Bias=30.5, SD of bias 82.18.
